# Significance of Twist expression and its association with E-cadherin in esophageal squamous cell carcinoma

**DOI:** 10.1186/1756-9966-28-158

**Published:** 2009-12-21

**Authors:** Ken Sasaki, Shoji Natsugoe, Sumiya Ishigami, Masataka Matsumoto, Hiroshi Okumura, Tetsuro Setoyama, Yasuto Uchikado, Yoshiaki Kita, Kiyokazu Tamotsu, Akihiko Sakamoto, Tetsuhiro Owaki, Takashi Aikou

**Affiliations:** 1 Department of Surgical Oncology and Digestive Surgery, Field of Oncology, Course of Advanced Therapeutics, Kagoshima University Graduate School of Medical and Dental Sciences, Sakuragaoka, Kagoshima, Japan

## Abstract

**Background:**

Twist is a basic helix-loop-helix (bHLH) transcriptional factor that has been identified to play an important role in epithelial-mesenchymal transition (EMT)-mediated metastasis through the regulation of E-cadherin expression. However, few authors have examined the expression of Twist and E-cadherin and their prognostic value in patients with esophageal squamous cell carcinoma (ESCC). The purpose of this study is to evaluate the clinical significance of Twist and E-cadherin expression in ESCC.

**Methods:**

We immunohistochemically investigated the relationship between their expression and clinicopathological factors including prognosis in surgical specimens of primary tumors in 166 patients with ESCC.

**Results:**

The expression rate of high Twist was 42.0% and that of preserved E-cadherin was 40.4%. The expression of high Twist and reduced E-cadherin was significantly associated with depth of tumor invasion, lymph node metastasis, distant nodal metastasis, stage and lymphatic invasion, and poor prognosis. High Twist expression significantly correlated with reduced E-cadherin expression. In the preserved E-cadherin group, the 5-year survival rate was better for patients who were low for Twist expression than for those who were high for Twist expression. Multivariate analysis indicated that the combination of low Twist and preserved E-cadherin expression was an independent prognostic factor along with tumor depth, distant nodal metastasis and E-cadherin expression.

**Conclusions:**

Evaluation of Twist and E-cadherin expressions should be useful for determining tumor properties, including prognosis, in patients with ESCC.

## Background

Epithelial-mesenchymal transition (EMT) is essential for morphogenesis during embryonic development and is a key event in the tumor invasion and metastatic processes [1]. E-cadherin, a homophilic Ca^2+^-dependent cell adhesion molecule located in adherens junctions of epithelia, plays a critical role in the suppression of tumor invasion; its loss of function coincides with increased tumor malignancy [2]. Several EMT-inducing regulators repress E-cadherin transcription via interaction with specific E-boxes of the proximal E-cadherin promoter [3]. Snail-related zinc finger transcription factors are the most prominent ones and we previously examined the relationship between E-cadherin and Snail or Slug expression in ESCC, close relationships were found [4,5]. Twist, a highly conserved basic helix-loop-helix (bHLH) transcription factor, has been recently identified as a developmental gene with a key role in E-cadherin repression and EMT induction [3]. There has not been any report on the association between Twist and E-cadherin expression in ESCC. The purpose of the present study was to examine the clinical significance of Twist expression in ESCC and the correlation between Twist and E-cadherin expression in ESCC.

## Methods

### Patients and specimens

The present study involved 166 patients with ESCC (149 men and 17 women) who underwent curative surgery at the Kagoshima University Hospital between January 1987 and December 1998. All patients underwent an esophagectomy with lymph node dissection. The patients ranged in age from 36 to 84 years (mean, 64.3 years). None of these patients underwent endoscopic mucosal resection, palliative resection, preoperative chemotherapy and/or radiotherapy, and none of them had synchronous or metachronous multiple cancers in other organs. Specimens of cancer and adjacent noncancerous tissues were collected from the patients according to the institutional guidelines of our hospital after informed consent had been obtained. Classifications of the specimens were determined according to the International Union Against Cancer tumor-node-metastasis classification system [6]. All of the M1 tumors had distant lymph node metastases. All patients were followed up after discharge with a chest X-ray every 1 to 3 months, computed tomography every 3 to 6 months, and ultrasonography every 6 months. Bronchoscopy and endoscopy were performed when necessary. Follow-up data after surgery were available for all patients with a median follow-up period of 24 months (range, 1-181 months).

### Immunohistochemical staining and evaluation

**Figure 1:**
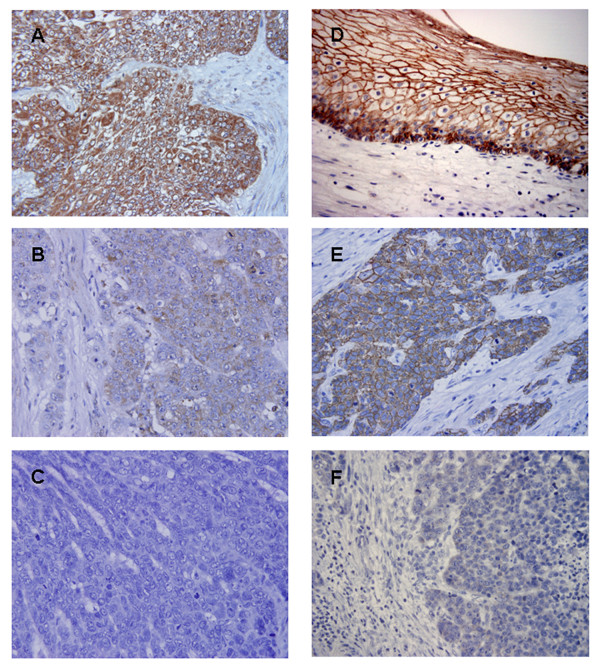
**Expression of Twist and E-cadherin proteins in ESCCs**. (**A**) High expression of Twist. (**B**) Weak expression of Twist. (**C**) Negative expression of Twist. (**D**) Preserved expression of E-cadherin is detected in the cancer adjacent normal tissue. (**E**) Preserved expression of E-cadherin. (**F**) Reduced expression of E-cadherin (Original magnification, ×400).

Tumor samples were fixed with 10% formalin in phosphate-buffered saline (PBS), embedded in paraffin, and sectioned into 3-μm slices. They were deparaffinized in xylene and dehydrated with a series of graded ethanol. For antigen retrieval, sections were heated in 10 mM citrate buffer solution for 15 minutes at 95°C for Twist and for 10 minutes at 120°C for E-cadherin, respectively. The endogenous peroxidase activity of specimens was blocked by immersing the slides in a 0.3% hydrogen peroxide (H_2_O_2_) solution in methanol for 30 minutes at room temperature. After washing three times with PBS for 5 minutes each, the sections were treated with 1% bovine serum albumin for 30 minutes to block nonspecific reactions at room temperature. The blocked sections were incubated with primary antibody Twist (Santa Cruz Biotechnology, Santa Cruz, CA; H-81, 1:100) or E-cadherin (Takara Biotechnology, Otsu City, Japan, 1:100), diluted in PBS at 4°C for overnight, followed by staining with a streptavidin-biotin peroxidase kit (Nichirei, Tokyo, Japan). The sections were washed in PBS for 5 minutes three times and the immune complex was visualized by incubating the sections with diaminobenzidine tetrahydrochloride. The sections were rinsed briefly in water, counterstained with hematoxylin, and mounted. Normal esophageal epithelium and invasive lobular carcinoma were used as positive controls for E-cadherin and Twist, respectively. Negative controls were created by replacing the primary antibodies with PBS. Evaluation of immunohistochemistry was independently carried out by two investigators (K.S. and I.S.) who were unaware of the clinical data or disease outcome. In cases in which the results of immunohistochemical expression differed between the two observers, slides were evaluated by a third observer (S.N.). For Twist, cytoplasmic immunoreactivity was scored by its extent and intensity. Staining intensity was graded as follows: negative (0), weak (1), moderate (2) and strong (3). Staining extent was rated according to the percentage of positive cells. Samples with no stained tumor cells were rated as 0, those with < 25% of stained tumor cells were rated as 1, those with 25-50% of stained tumor cells were rated as 2, those with 50-75% of stained tumor cells were rated as 3 and those with > 75% of stained tumor cells were rated as 4. The results of staining intensity and extent gave an overall staining score. An overall staining score of 0-5 and 6-7 were regarded as low and high Twist expression, respectively. For E-cadherin, cancer cells were divided into two groups: preserved expression, which indicates cells with the same level of expression as that of normal epithelium distant enough from tumor, and reduced expression, which indicates cells with weak or absent expression compared with normal epithelium (Fig. 1) [7]. To evaluate expression of Twist and E-cadherin, ten fields (within the tumor and at the invasive front) were selected and expression in 1000 tumor cells (100 cells/field) was evaluated using high-power (×200) microscopy.

### Statistical analysis

Statistical analysis of group differences was done using the X^2 ^and Wilcoxon tests. The Kaplan-Meier method was used for survival analysis and differences in survival were estimated using the log-rank test. Prognostic factors were examined by univariate and multivariate analyses (Cox proportional hazards regression model). *P *< 0.05 was considered to be statistically significant. All statistical analyses were done with the software package JMP 5 for Windows (SAS Institute, Inc., Cary, NC).

## Results

### Expressions of Twist and E-cadherin in esophageal squamous cell carcinoma

Twist expression was observed in the cytoplasm of cancer cells in 42.0% of all patients (70 of 166; Fig. 1A). E-cadherin expression was observed on the cell membrane of cancer cells, indicating preserved expression, in 40.4% of all patients (67 of 166; Fig. 1B).

### Relationship between the expressions of Twist and E-cadherin and clinicopathological findings

The expression of Twist and E-cadherin was significantly associated with the following clinicopathological parameters: depth of tumor invasion, lymph node metastasis, distant nodal metastasis, stage and lymphatic invasion. Tumors with high Twist expression invaded deeper (*P *= 0.0044), had more lymph node metastasis (*P *= 0.038), had more distant nodal metastasis (*P *= 0.0073), had a more advanced stage (*P *= 0.0011) and had more lymphatic invasion (*P *= 0.0011) than those that were low Twist expression. Tumors with reduced E-cadherin expression invaded deeper (*P *< 0.0001), had more lymph node metastasis (*P *< 0.0001), had more distant nodal metastasis (*P *= 0.002), had a more advanced stage (*P *< 0.0001) and had more lymphatic invasion (*P *= 0.0008) than those that were preserved E-cadherin expression. Presence of high Twist expression significantly correlated with reduced E-cadherin expression (*P *= 0.0076) (Table 1).

**Table 1:** Twist and E-cadherin expression in relation to clinicopathological findings

		Twist		***P***	E-cadherin		***P***
					
	Total (*n *= 166)	High	Low		Preserved	Reduced	
					
		*n *= 70 (40.2%)	*n *= 96 (57.8%)		*n *=67 (40.4%)	*n *=99 (59.6%)	
**Age**		65.1 ± 9.0	63.7 ± 9.4	0.52	63.6 ± 9.8	64.8 ± 8.9	0.70
**Sex**							
**Male**	149 (89.8)	63 (90.0)	86 (89.6)	0.93	59 (88.1)	90 (90.9)	0.56
**Female**	17 (10.2)	7 (10.0)	10 (10.4)		8 (11.9)	9 (9.1)	
**Tumor location**							
**Upper**	28 (16.9)	16 (22.9)	12 (12.5)	0.21	13 (19.4)	15 (15.2)	0.70
**Middle**	76 (45.8)	29 (41.4)	47 (49.0)		31 (46.3)	45 (45.5)	
**Lower**	62 (37.4)	25 (35.7)	37 (38.5)		23 (34.3)	39 (39.4)	
**Histology**							
**Well**	63 (38.0)	31 (44.3)	32 (33.3)	0.26	24 (35.8)	39 (39.4)	0.13
**Moderate**	76 (45.8)	27 (38.6)	49 (51.0)		36 (53.7)	40 (40.4)	
**Poor**	27 (16.3)	12 (17.1)	15 (15.6)		7 (10.5)	20 (20.2)	
**pT**							
**pT1**	46 (27.7)	10 (14.3)	36 (37.5)	0.0044	33 (49.3)	13 (13.1)	<.0001
**pT2**	25 (15.1)	10 (14.3)	15 (15.6)		11 (16.4)	14 (14.1)	
**pT3**	67 (40.4)	34 (48.6)	33 (34.4)		14 (20.9)	53 (53.5)	
**pT4**	28 (16.9)	16 (22.9)	12 (12.5)		9 (13.4)	19 (19.2)	
**pN**							
**pN0**	65 (39.2)	21 (30.0)	44 (45.8)	0.038	44 (65.7)	21 (21.2)	<.0001
**pN1**	101 (60.8)	49 (70.0)	52 (54.2)		23 (34.3)	78 (78.8)	
**pM**							
**pM0**	118 (71.1)	42 (60.0)	76 (79.2)	0.0073	58 (86.6)	60 (60.6)	0.0002
**pM1**	48 (28.9)	28 (40.0)	20 (20.6)		9 (13.4)	39 (39.4)	
**pStage**							
**I**	30 (18.1)	7 (10.0)	23 (24.0)	0.0011	26 (38.8)	4 (4.0)	<.0001
**IIA**	29 (17.5)	10 (14.3)	19 (19.8)		15 (22.4)	14 (14.1)	
**IIB**	21 (12.7)	4 (5.7)	17 (17.7)		10 (14.9)	11 (11.1)	
**III**	38 (22.9)	21 (30.0)	17 (17.7)		7 (10.5)	31 (31.1)	
**IV**	48 (28.9)	28 (40.0)	20 (20.8)		9 (13.4)	39 (39.4)	
**Lymphatic invasion**							
**Positive**	107 (64.5)	55 (78.6)	52 (54.2)	0.0010	33 (49.3)	74 (74.8)	0.0008
**Negative**	59 (35.5)	15 (21.4)	44 (45.8)		34 (50.8)	25 (25.3)	
**Venous invasion**							
**Positeive**	51 (30.7)	26 (37.1)	25 (26.0)	0.13	17 (25.4)	34 (34.3)	0.22
**Negative**	115 (69.3)	44 (62.9)	71 (74.0)		50 (74.6)	65 (65.7)	
**E-cadherin expression**							
**Preserved**	67 (40.4)	20 (28.6)	47 (49.0)	0.0076			
**Reduced**	99 (59.6)	50 (71.4)	49 (51.0)				

### Relationship between Twist expression and clinicopathological findings according to E-cadherin expression

The tumors were divided into the preserved E-cadherin group and reduced E-cadherin group. In the E-cadherin preserved group, the expression of Twist was related to lymphatic invasion; in the E-cadherin reduced group, the expression of Twist was related to depth of tumor invasion and stage (Table 2).

**Table 2:** Relationship between Twist expression and clinicopathological findings according to E-cadherin expression

	E-cadherin preserved		***P***	E-cadherin reduced		*P*
				
Characteristics	Twist high	Twist low		Twist high	Twist low	
				
	*n *= 20 (29.9%)	*n *= 47 (70.2%)		*n*=50 (50.5%)	*n *= 49 (49.5%)	
**Histology**						
**Well**	7 (35.0)	17 (36.2)	0.74	24 (48.0)	15 (30.6)	0.20
**Moderate**	10 (50.0)	26 (55.3)		17 (34.0)	23 (46.9)	
**Poor**	3 (15.0)	4 (8.5)		9 (18.0)	11 (22.5)	
**pT**						
**pT1**	8 (40.0)	25 (53.2)	0.28	2 (4.0)	11 (22.5)	0.027
**pT2**	4 (20.0)	7 (14.9)		6 (12.0)	8 (16.3)	
**pT3**	3 (15.0)	11 (23.4)		31 (62.0)	22 (44.9)	
**pT4**	5 (25.0)	4 (8.5)		11 (22.0)	8 (16.3)	
**pN**						
**pN0**	10 (50.0)	34 (72.3)	0.082	11 (22.0)	10 (20.4)	0.85
**pN1**	10 (50.0)	13 (27.7)		39 (78.0)	39 (79.6)	
**pM**						
**pM0**	16 (80.0)	42 (89.4)	0.32	26 (52.0)	34 (69.4)	0.076
**pM1**	4 (20.0)	5 (10.6)		24 (48.0)	15 (30.6)	
**pStage**						
**I**	7 (35.0)	19 (40.4)	0.24	0 (0.0)	4 (8.2)	0.0022
**IIA**	2 (10.0)	13 (27.7)		8 (16.0)	6 (12.2)	
**IIB**	3 (15.0)	7 (14.9)		1 (2.0)	10 (20.4)	
**III**	4 (20.0)	3 (6.4)		17 (34.0)	14 (28.6)	
**IV**	4 (20.0)	5 (10.6)		24 (48.0)	15 (30.6)	
**Lymphatic invasion**						
**Positive**	14 (70.0)	19 (40.4)	0.025	41 (82.0)	33 (67.4)	0.092
**Negative**	6 (30.0)	28 (59.6)		9 (18.0)	16 (32.7)	
**Venous invasion**						
**Positeive**	8 (40.0)	9 (19.2)	0.080	18 (36.0)	16 (32.7)	0.73
**Negative**	12 (60.0)	38 (80.9)		32 (64.0)	33 (67.4)	

### Relationship between prognosis and expression of Twist and E-cadherin

**Figure 2:**
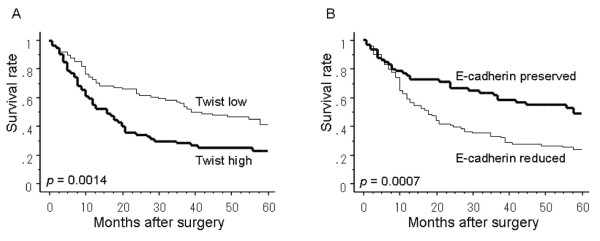
**The postoperative 5-year survival curve of patients according to the expression of Twist (A) and E-cadherin (B) proteins**. There was a significant difference in survival between the patients with high and low expressions of Twist (*P *= 0.0014). There was also a significant difference in survival between the patients with preserved and reduced expressions of E-cadherin (*P *= 0.0007).

Seven of the patients died of postoperative complications within 30 days of the beginning of the study period, leaving 159 patients for survival analysis. The 5-year survival rate of patients with tumors with low and high Twist expression was 41.6%, whereas the rate for high Twist expression was 23.0%.There was a significant difference in 5-year survival rate between low and high expression of Twist (*P *= 0.0014; Fig. 2A). The 5-year survival rate of patients with tumors with preserved and reduced E-cadherin expression was 48.7% and 23.3%, respectively, and the difference was significant (*P *= 0.0007; Fig. 2B).

### Relationship between prognosis and Twist expression in the preserved and reduced E-cadherin groups

**Figure 3:**
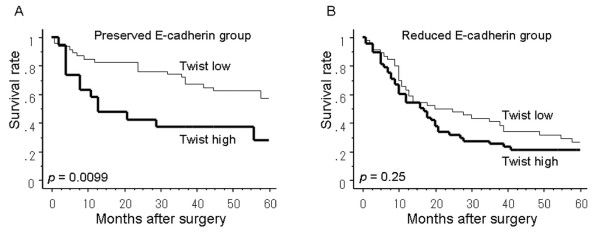
**The postoperative 5-year survival curves between the patients with high Twist or low Twist expression according to E-cadherin expressions**. (**A**) In the preserved E-cadherin group, the patients with low Twist expression had a better outcome than those with high Twist expression (*P *= 0.0099). (**B**) In the reduced E-cadherin group, the survival curve was not significantly different according to the Twist expression (*P *= 0.25).

**Figure 4:**
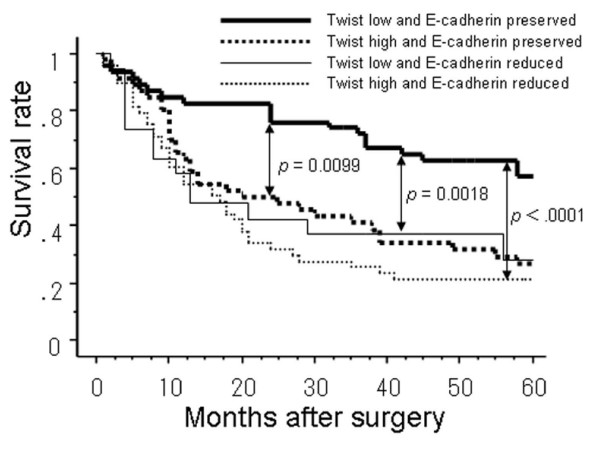
**The postoperative 5-year survival curves according to combined expression of Twist and E-cadherin**. Five-year survival rate of patients with both low Twist and preserved E-cadherin expression had a significant better outcome than those with the other groups.

In the preserved E-cadherin group, the 5-years survival rate was significantly higher for patients low for Twist expression than for those high for Twist expression (*P *= 0.0099; Fig. 3A). However, in the E-cadherin reduced group, there was no significant difference between patients high and low for Twist expression (Fig. 3B). Moreover, the 5-years survival rate was significantly worse in patients with high Twist and reduced E-cadherin expression tumors than those with low Twist and preserved E-cadherin expression (*P *< 0.0001; Fig. 4).

### Univariate and multivariate analyses of survival

Univariate analysis showed that the following factors were significantly related to postoperative survival: tumor depth, lymph node metastasis, distant metastasis, stage, lymphatic invasion, venous invasion, Twist expression, E-cadherin expression and the combination of Twsit and E-cadherin expression (*P *< 0.05). Multivariate regression analysis indicated that depth of invasion, distant metastasis, E-cadherin expression and the combination of Twsit and E-cadherin expression were independent prognostic factors (Table 3).

**Table 3:** Univariate and multivariate analyses of prognostic factors

Independent factors	Univariate *P*	Multivariate *P*	Hazard ratio	95% confidence interval
**pT**				
**(pT1, 2/pT3, 4)**	<.0001	<.0001	2.767	1.734-4.526
**pN**				
**(pN0/pN1)**	<.0001	0.1490	1.588	0.848-3.006
**pM**				
**(pM0/pM1)**	<.0001	0.0042	2.013	1.247-3.278
**Lymphatic invasion**				
**(Negative/Positive)**	0.0001	0.6098	1.159	0.661-2.060
**Venous invasion**				
**(Negative/Positive)**	0.0057	0.6879	1.094	0.704-1.690
**Twist**				
**(Low/High)**	0.0021	0.6635	0.898	0.554-1.465
**E-cadherin**				
**(Preserved/Reduced)**	0.0007	0.0307	2.247	1.083-4.424
**Combination of Twist and E-cadherin**				
**(Twist low + E-cadherin preserved/other groups)**	<.0001	0.0371	2.547	1.059-5.986

## Discussion

Ectopic expression of Twist results in loss of E-cadherin-mediated cell-cell adhesion, activation of mesenchymal markers, and induction of cell motility, suggesting that Twist contributes to tumor invasiveness, metastasis and prognosis by promoting an EMT [3]. Recently, up-regulation of Twist has been reported in several types of human cancer [3,8-12]. The rates of high Twist and reduced E-cadherin expression have been reported as 36-60% and 44-74%, respectively [12-17].

In our present investigation, immunohistochemistry demonstrated that rates of high Twist and reduced E-cadherin expression were 42.0 and 40.4%. Upregulation of Twist [14] expression has been associated with high incidence of distant metastasis and downregulation of E-cadherin [15,18] expression has been associated with high incidence of lymph node metastasis in ESCC. In this study, depth of tumor invasion, lymph node metastasis, distant nodal metastasis, stage and lymphatic invasion were significantly associated with high Twist or reduced E-cadherin expression.

Additionally, presence of high Twist expression significantly correlated with reduced E-cadherin expression. Inverse correlation between high Twist and reduced E-cadherin expression has been found in liver, endometrial, bladder and prostate human cancer cells [12,13,19,20]. Thus, the present results are almost consistent with previous reports. Prognosis was poorer in patients with high Twist expression than in those with low Twist expression. Similarly, the prognosis was worse in patients with reduced E-cadherin than those in with preserved E-cadherin expression, which agrees with previous reports. The patients with both low Twist and preserved E-cadherin expression had the best clinical outcome according to univariate analysis and it was an independent prognostic factor on multivariate analysis. On the strength of these data, we speculate that high Twist expression may promote EMT by dysregulation of the E-cadherin expression pattern in ESCC.

However, some patients with preserved E-cadherin expression had poor prognosis. In the preserved E-cadherin group, the patients were high for Twist expression had more lymphatic invasion and worse prognosis. Thus, it seems that Twist not only suppresses the function of E-cadherin but also promotes lymphatic invasion in the preserved E-cadherin group and several hypotheses might explain. Twist has been recently identified as a developmental gene with a key role in E-cadherin repression and EMT induction. Twist gene is also a newly-know potential oncogene and metastasis related gene [3,21]. Twist can inhibit myc oncogene- and p53-dependent apoptosis in mouse embryonic fibroblasts [21] and NF-κB pathway dependent apoptosis [22]. It also suppresses cellular differentiation and protects apoptosis through inhibition of p21^WAF1/Cip1^, inhibitor of cyclin-dependent kinases, via both p53-dependent and independent pathways [23]. Mesenchyme Forkhead 1 (FOXC2) which induced by Twist, Snail, Goosecoid and TGF-β1 plays a central role in promoting invasion and metastasis in human basal-like breast cancers [24]. Twist expression is regulated by Wnt/β-catenin signaling and that both Wnt and Twist can function as inhibitors of lactogenic differentiation, an effect that could contribute to mammary tumorigenesis [25]. These reports might explain the aggressive behavior of the patients with high Twist expression.

Snail, Slug and Twist are transcriptional factors that regulate the expression of E-cadherin. We have previously studied the expression of Snail [4], Slug [5] and Twist [this study] in ESCC patients. Subjects were 194, 206 and 166, respectively, of which 110 were shared subjects. We reexamined the correlation of the expression of Snail, Slug, Twist and E-cadherin in 110 ESCC patients. The expression of Twist was significantly associated with Snail, Slug and E-cadherin, respectively (*P *= 0.0266, *P *= 0.0137 and *P *= 0.0024). The univariate analyses of combination of Twist and Snail expression (Twist negative + Snail negative/others) and Twist and Slug expression (Twist negative + Slug negative/others) showed significantly correlation, respectively (*P *= 0.0015 and *P *= 0.0017). These data demonstrated that all three transcription factors have inappropriate expressed in ESCC and these factors are significantly correlated with each other.

## Conclusions

Twist or E-cadherin expression was associated with tumor properties, including depth of tumor invasion, lymph node metastasis, distant nodal metastasis, stage, lymphatic invasion and prognosis. Evaluation of Twist and/or E-cadherin expression is useful for determining malignant properties, including clinical outcome in patients with ESCC.

## Competing interests

The authors declare that they have no competing interests.

## Authors' contributions

All the authors contributed as mentioned. KS and SN conceived of the study and drafted the manuscript. SI, MM, HO, TS, YU, YK, KT, AS, and TO participated in designing the study and helped to write the paper. TA supervised the entire study. All authors have read and approved the final manuscript.
